# Nonatopic Eosinophilic Duodenitis in an Adult: A Case Report and Overview

**DOI:** 10.7759/cureus.43919

**Published:** 2023-08-22

**Authors:** Davong D Phrathep, Marc R Mohammed, Andrew B Herson, Kevin D Healey, Ali El-Husari, Michael Herman

**Affiliations:** 1 College of Osteopathic Medicine, Lake Erie College of Osteopathic Medicine, Bradenton, USA; 2 Medicine, Touro College of Osteopathic Medicine, Harlem, USA; 3 Physical Medicine and Rehabilitation, Lake Erie College of Osteopathic Medicine, Bradenton, USA; 4 Gastroenterology, Borland Groover, Jacksonville, USA

**Keywords:** recurrent abdominal pain, atopic, eosinophilic gastrointestinal disorders, abnormal rise of eosinophils, eosinophilic duodenitis

## Abstract

Eosinophilic duodenitis is an inflammation of the duodenum, characterized by an abundance of eosinophils, typically triggered by hypersensitivity reactions. Typically, recurrent abdominal pain with eosinophilic duodenitis is rare in individuals without a history of atopic conditions like asthma. Here, we present the case of a 62-year-old man who experienced recurrent upper abdominal pain for 12 months and unintended weight loss for the past six months. The patient reported no allergies to food, drugs, or the environment, and has no history of other atopic conditions. Esophagogastroduodenoscopy (EGD) with biopsy of the duodenum and stomach revealed 32 eosinophils per high-power field (HPF), which is mild. Skin prick testing yielded negative results. Following initial treatment with H2 inhibitors, proton pump inhibitors, and budesonide for a total of 12 weeks, the patient reported an improvement in symptoms and subsequent weight gain. This report emphasizes a rare case of eosinophilic duodenitis in a nonatopic individual with a successful treatment regimen. His quality of life improved with weight gain, resolved abdominal pain, and improved appetite. Although the patient’s condition lasted about 12 months, our report showcased the importance of timely clinical diagnosis and appropriate combination therapy to alleviate progressive pain associated with eosinophilic duodenitis.

## Introduction

Eosinophils are white blood cells (WBCs) that are involved in innate and adaptive immunity [1]. On hematoxylin and eosin (H&E), eosinophils appear as round- or oval-shaped granules with a uniform texture and are bright red or orange. Initially, eosinophils were described as having an association with the immune response against parasitic infections; however, eosinophils play more roles in the human body, including tissue repair. Because eosinophils are typically involved in the immune defense against parasitic infections, they are not often found in high numbers in the gut mucosa of healthy patients [2]. In the absence of specific triggers for eosinophils in mucosal tissue, primary infiltrative eosinophilic disorders of the gut do occur and can present similarly to other gastrointestinal (GI) conditions [3]. This being said, when eosinophils are found in large numbers in the gut, particular diagnoses need to be further explored and worked up. Currently, the cause of mucosal eosinophilia is not completely understood. However, there are a variety of conditions that have been linked with mucosal eosinophilia such as autoimmune disorders, drug reactions, inflammatory bowel disorders, bacterial infections, parasitic infections, and food allergies [4]. In recent years, there has been increasing research about mucosal eosinophilia, particularly eosinophilic duodenitis. Eosinophilic GI disease, specifically eosinophilic duodenitis, is a chronic inflammatory condition that is characterized by elevated levels of eosinophils in the GI tract and causes persistent GI symptoms [5].

## Case presentation

A 62-year-old Caucasian male with a past medical history of hypertension presented to the outpatient clinic for evaluation of persistent abdominal pain and loose stools for 12 months. The patient reported that his abdominal symptoms worsened after meals but denied any associated nausea or vomiting. Over the past six months, he had experienced 8 pounds of unintended weight loss. He reported no recent changes in his lisinopril dosage for his hypertension and reported no recent travel. The patient reported no alcohol or tobacco use and no history of any known food, drug, or environmental allergies. He had no history of surgeries. Vital signs at the clinic showed a blood pressure of 142/84 mmHg, pulse of 68 beats/minute, temperature of 36.7 °C, respiratory rate of 18 breaths/minute, and O2 saturation of 98%.

On examination, the patient presented with upper abdominal tenderness, but no other remarkable findings were noted. Stool studies, including ova, parasites, and cultures, were negative. Laboratory investigations revealed a normal differential, a WBC count of 5, a hemoglobin (Hgb) level of 13, and normal results on the complete metabolic panel, as shown in Table 1.

**Table 1:** Complete metabolic panel values.

Complete metabolic panel		
Glucose	96 mg/dL	65-99 mg/dL
Urea nitrogen	15 mg/dL	7-25 mg/dL
Creatinine	0.95 mg/dL	0.7-1.33 mg/dL
Sodium	137 mmol/L	135-146 mmol/L
Potassium	4.3 mmol/L	3.5-5.3 mmol/L
Chloride	104 mmol/L	98-110 mmol/L
Calcium	9.4 mg/dL	8.6-10.3 mg/dL
Total protein	7.3 g/dL	6.1-8.1 g/dL
Alkaline phosphotase	95 units/L	35-144 units/L
Aspartate aminotransferase	23 units/L	10-35 units/L
Alanine aminotransferase	24 units/L	9-46 units/L

Additionally, a CT scan of the abdomen was unremarkable. The initial treatment comprised a three-week course of H2 blockers, which was subsequently switched to proton pump inhibitors (PPI). While this led to a mild improvement in the patient's abdominal discomfort, there was no notable alteration in the loose stools or weight loss.

**Figure 1:**
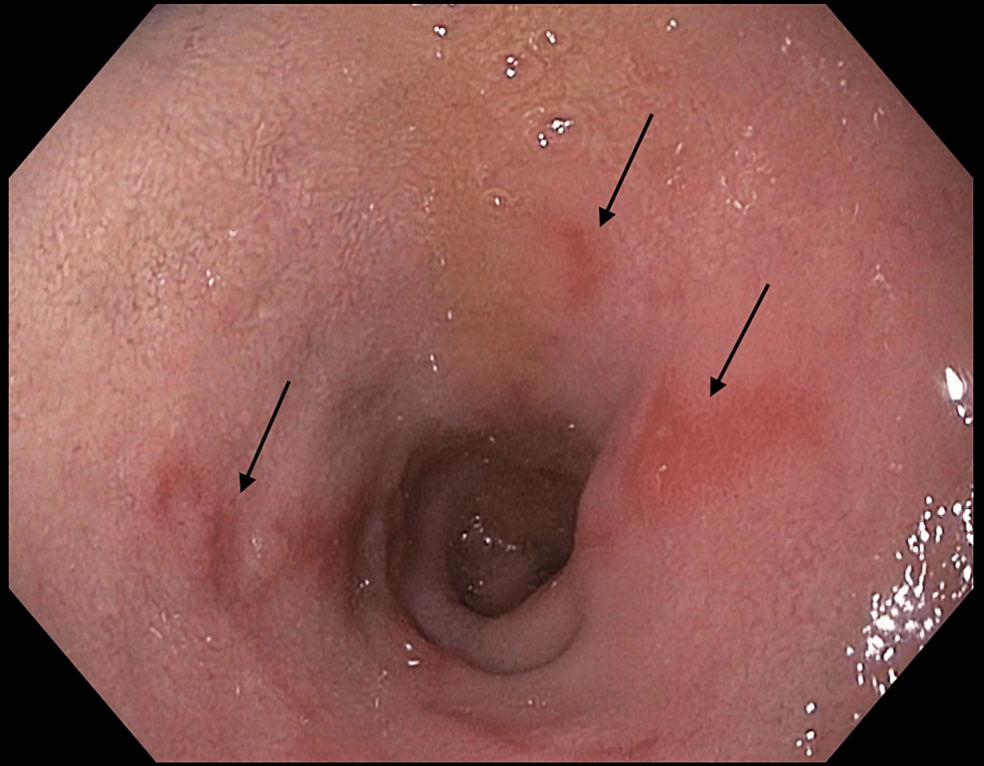
Erythematous patches in the duodenum.

Further evaluation via esophagogastroduodenoscopy (EGD) was arranged for symptom evaluation. The EGD revealed areas of patchy erythema in the duodenum, including multiple erythematous patches (Figure 1).

Multiple biopsies were obtained. No evidence of *Helicobacter pylori* infection was found. The pathology report was consistent with eosinophilic duodenitis and gastritis, revealing 32 eosinophils/high-power fields (HPFs) and mucosal layer involvement. Skin prick testing for allergies was negative. Based on the patient's clinical presentation, investigative findings, and EGD results, the diagnosis of eosinophilic duodenitis and gastritis was made. The patient was referred to a gastroenterologist for additional evaluation and management. He was treated with budesonide 9 mg once a day for four weeks. He was then placed on budesonide 6 mg once a day for four weeks and then subsequently 3 mg once a day for four weeks. This treatment regimen resulted in the resolution of his symptoms and subsequent weight gain.

## Discussion

Eosinophilic gastrointestinal diseases (EGIDs) is a term used for chronic, immune-mediated conditions characterized clinically by GI symptoms and histologically by pathologic infiltration of the GI tract by eosinophils. EGIDs are classified by the site of eosinophilic infiltration. Eosinophilic duodenitis is defined as eosinophilia of the duodenum [6]. Eosinophilic gastrointestinal diseases appear in all age ranges, but the literature suggests that most patients are adults. The cause of eosinophilic duodenitis is unknown, however, previous studies have indicated that patients with eosinophilic duodenitis often have comorbid allergic diseases, such as asthma, food allergy, or peripheral eosinophilia [7].

Although epidemiological data are limited for eosinophilic duodenitis in the United States, it is believed that the estimated prevalence for eosinophilic duodenitis is 8.4/100,000 [8]. According to data collected by Jensen et al., the female predominance for the disease was most evident for eosinophilic gastritis at 7.9 patients/100,000 when compared to 5.4 patients/100,000 for males [7]. Regarding regional differences among eosinophilic gastritis and gastroenteritis patients in the United States, the South and Midwest had nearly twice the prevalence than those in the Northeast and West [7]. When present, the most commonly reported symptoms of eosinophilic duodenitis and gastritis are nausea/vomiting and abdominal pain. Other gastrointestinal symptoms reported by patients with eosinophilic duodenitis and gastritis are diarrhea and early satiety [8]. 

Eosinophilic duodenitis, like other eosinophilic gastrointestinal disorders, has traditionally been associated with allergic and immune-mediated mechanisms. This condition has been less common in patients without allergies in comparison to those with allergies. The exact prevalence of eosinophilic duodenitis in the absence of allergies is not well-defined and research on this specific subgroup is still emerging in the literature. Other factors, including genetic predisposition, immune dysregulation, and interactions between environmental and genetic factors, may contribute to the development of eosinophilic duodenitis. Our case provides additional research evidence of eosinophilic duodenitis and contributes to the limited reports in patients without allergies and other predisposed factors for the condition.

Unlike eosinophilic esophagitis, there are no consensus guidelines for the diagnosis of eosinophilic GI disease. The gold standard for diagnosis is endoscopy and observation of a large number of eosinophils in one HPF on histopathologic evaluation of biopsies [9]. Currently, the Food and Drug Administration (FDA) has accepted >30 eosinophils per HPF in >3 HPF in the duodenum as the threshold values for diagnosis of eosinophilic duodenitis [5]. To obtain the ideal biopsy specimen, one study suggested that the observer should focus on locating fields with the highest eosinophil density and within the lamina propria. In the stomach, mild increases (30 to 50 eosinophils/HPF) and moderate increases (50 to 80 eosinophils/HPF) have been associated with insignificant mucosal changes [5]. However, dense eosinophilic infiltrates (80 to 100 eosinophils/HPF) have been associated with surface epithelial damage, mucin depletion, other inflammatory infiltrates, and foveolar hyperplasia [5]. 

Some authors have proposed strict definitions of countable eosinophils, including cells filled with eosinophilic granules and displaying both visible lobes of the nucleus [10]. In the same study, countable eosinophilia was defined as a cell that presents one of the three distinct appearances: a partial nucleus, a bilobed nucleus, or a discrete cluster of eosinophil granules, at least partially enclosed by a membrane, even if a discernible nucleus is absent [5]. Counting cells without the aid of a counting device is feasible for small numbers. In this case, observers often use subitizing, which allows them to accurately and quickly assess the number of eosinophils in a sample [11]. Emerging evidence suggests that a mean count of 20 eosinophils/HPF in gastric biopsy specimens or 30 eosinophils/HPF in duodenal biopsy specimens may effectively identify patients with high specificity [12]. As cell counts increase in number beyond 10 to 20 cells/HPF, memorizing the numbers and mentally adding them up can become cumbersome and confusing. Therefore, observers may opt into using a mechanical cell counter. Topical and systemic glucocorticoids are used as the current standard of care and have been reported to reduce symptoms, but their long-term use is limited by toxicity [13]. Although there has not been a consistent finding in symptom resolution with the off-label use of biologics, biologics targeting IgE, the IL-5 pathway, or integrins have been shown to reduce tissue eosinophilia [13]. Eosinophilic esophagitis shares a similar pathogenesis but is limited to the esophagus [14]. Eosinophilic duodenitis is marked by high levels of eosinophils in the gastrointestinal mucosa. The involvement of mast-cell activity in the conditions' development is suspected. AK002 (lirentelimab), an anti-Siglec-8 antibody, has demonstrated promise in animal models as a potential treatment for these conditions by depleting eosinophils and inhibiting mast cells [13].

## Conclusions

Recurrent abdominal pain associated with eosinophilic duodenitis is relatively uncommon in individuals without a history of atopic conditions, such as asthma. We present a unique case of a 62-year-old man who experienced recurring upper abdominal pain and unintentional weight loss. Our patient had no reported allergies to food, drugs, or environmental factors and had no previous history of atopic conditions. Skin prick testing yielded negative results, further ruling out allergens as potential triggers. For diagnostic purposes, our report mentions the importance of EGD and described the current approach to interpreting biopsy results in the setting of eosinophilic duodenitis. Encouragingly, the patient reported an improvement in symptoms with regained lost weight following our treatment regimen. This report highlights a case of eosinophilic duodenitis occurring in a nonatopic individual, with a successful and timely treatment approach. Our findings provide an overview of eosinophilic duodenitis and underscore the importance of prompt clinical diagnosis and the significance of appropriate combination therapy in alleviating progressive symptoms associated with the condition.
